# Effect of Hydroxyapatite Coating by Er: YAG Pulsed Laser Deposition on the Bone Formation Efficacy by Polycaprolactone Porous Scaffold

**DOI:** 10.3390/ijms23169048

**Published:** 2022-08-12

**Authors:** Ye Zhang, Jun-Ichiro Jo, Liji Chen, Shigeki Hontsu, Yoshiya Hashimoto

**Affiliations:** 1 Department of Biomaterials, Osaka Dental University, 8-1 Kuzuhahanazonocho, Hirakata-shi, Osaka 573-1121, Japan; 2 Department of Orthodontics, Osaka Dental University, 8-1 Kuzuhahanazonocho, Hirakata-shi, Osaka 573-1121, Japan; 3 Department of Biomedical Engineering, Faculty of Biology-Oriented Science and Technology, Kindai University, 930 Nishimitani, Kinokawa-shi, Wakayama 649-6493, Japan

**Keywords:** hydroxyapatite coating, Er: YAG laser, pulsed laser deposition, polycaprolactone, porous scaffold, bone formation

## Abstract

Composite scaffolds obtained by the combination of biodegradable porous scaffolds and hydroxyapatite with bone regeneration potential are feasible materials for bone tissue engineering. However, most composite scaffolds have been fabricated by complicated procedures or under thermally harsh conditions. We have previously demonstrated that hydroxyapatite coating onto various substrates under a thermally mild condition was achieved by erbium-doped yttrium aluminum garnet (Er: YAG) pulsed laser deposition (PLD). The purpose of this study was to prepare a polycaprolactone (PCL) porous scaffold coated with the hydroxyapatite by the Er: YAG-PLD method. Hydroxyapatite coating by the Er: YAG-PLD method was confirmed by morphology, crystallographic analysis, and surface chemical characterization studies. When cultured on PCL porous scaffold coated with hydroxyapatite, rat bone marrow-derived mesenchymal stem cells adhered, spread, and proliferated well. The micro-CT and staining analyses after the implantation of scaffold into the critical-sized calvaria bone defect in rats indicate that PCL porous scaffold coated with hydroxyapatite demonstrates accelerated and widespread bone formation. In conclusion, PCL porous scaffold coated with hydroxyapatite obtained by the Er: YAG-PLD method is a promising material in bone tissue engineering.

## 1. Introduction

Loss of alveolar bone caused by innate diseases such as cleft lip and palate or periodontal disease, etc., is one of the issues to be resolved in the dental field. Under the circumstance, trials on the regeneration of alveolar bone have been performed by various approaches. Among the approaches, the implantation of autogenous bone has been recognized as a promising method for creating a good alveolar morphology and tooth eruption without disturbing maxillary jaw development [1]. Furthermore, it has been reported that the fresh autogenous bone has superior tissue stability and osteogenic potential compared to other materials, and that ectopic osteogenic potential exists in autogenous bone marrow [2]. On the other hand, there are reports that the autologous bone resorbs prematurely in clinical practice, resulting in no long-term effect [3]. Moreover, the high morbidity of the donor area and the dramatic decrease in healing success when the defect length is greater than 6 cm are not negligible [1,2,4]. In addition, the limited availability of autologous tissue, combined with extremely individualized and complex morphology, makes the craniomaxillofacial bone tissue repair very challenging [5]. In recent years, composite scaffolds that mimic the structure and biological function of healthy bone tissue in terms of chemical composition, hierarchical structure, and properties by integrating a three-dimensional printing technology applicable to biomaterials have gained widespread attention in the bone regenerative medicine community [6,7,8,9,10,11,12]. The composite scaffolds are advantageous over the autologous bone implantation in terms of non-invasiveness by the collection of host bone tissue, availability of application for the bone with individualized and complexed morphology, and no limitation of defect size by adding porous structure.

Polycaprolactone (PCL) is a biodegradable polymer used in many FDA-approved implants, drug delivery devices, and sutures, as well as in a wide range of applications in tissue engineering research [13,14,15,16,17]. The three-dimensional PCL porous scaffold is a non-cytotoxic bioreactor manufactured by a solvent-free manufacturing process, and its pores are 100% open, allowing for easy exchange of nutrients and cellular metabolic waste. In addition, the PCL scaffold material uses three-dimensional precision fine processing technology to obtain uniform fiber diameters and fine pore diameters, ensuring reproducible porous structures. Above all, the possibility of customizing the shape to fit the defect, the possession of moderate mechanical properties [18], and the resorbability that does not disturb the natural tissue growth [19] make it a possibility in the repair of bone loss. However, the no-bone regenerative potential of PCL itself [18] limits its usage for bone tissue engineering. Therefore, it is necessary to combine the PCL porous scaffold with compounds that possess a bone regenerative potential.

One of the plausible methods to meet the requirement is a coating of a hydroxyapatite, which is the main component of natural bone and has a high biological activity and safety [20,21]. There are several techniques of hydroxyapatite coating, which include sol-gel method, plasma spraying, electrophoretic deposition, electrochemical deposition, and biomimetic coating [22]. In the dental field, dental implants with which titanium and other inorganic material surfaces are coated by plasma spraying have been commercialized so far [23,24]. In recent years, pulsed laser ablation has been attracting attention as a method to obtain thin films of high-quality hydroxyapatite [25,26]. The advantages of pulse laser deposition over plasma spraying include a thin and uniform coating of hydroxyapatite without any sandblasting pre-treatment, the strong adhesive force of the substrate, and the toughness of coated hydroxyapatite [27]. The lasers used to fabricate hydroxyapatite thin films include excimer lasers such as ArF, KrF, XeCl, and XeF, and the third harmonic of Nd: yttrium aluminum garnet (YAG) lasers [28]. However, the coating of organic materials, especially biopolymer materials, should be performed at low temperature due to the lack of thermal tolerance. Several studies have previously demonstrated the successful deposition of α-tricalcium phosphate (α-TCP) on titanium plates or bovine enamel surfaces using Er: YAG pulsed laser deposition (PLD) and the conversion of α-TCP to calcium-deficient hydroxyapatite through hydrolysis reaction under a mild condition [29,30,31,32]. In this context, we conceived the usage of a dental Er: YAG laser unit [33] for the coating of hydroxyapatite onto a PCL porous scaffold.

The aim of this study was to prepare a PCL porous scaffold coated with a hydroxyapatite by the Er: YAG pulsed laser deposition (PLD) method and to evaluate the feasibility for the induction of bone formation. Hydroxyapatite coating on the PCL porous scaffold was confirmed, and the physical properties, biocompatibility, and bone regeneration ability were evaluated in vivo and in vitro.

## 2. Results

### 2.1. Hydroxyapatite Coating onto the PCL Porous Scaffold

**Figure 1:**
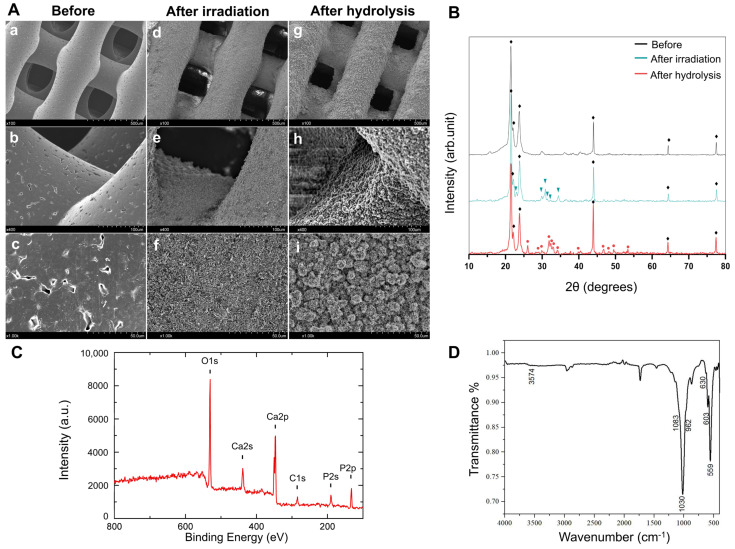
Characterization of hydroxyapatite coating onto PCL porous scaffold by Er: YAG PLD method. (**A**) SEM images of PCL porous scaffold before (**a**–**c**) and after (**d**–**f**) treatments of irradiation and hydrolysis (**g**–**i**). The images were taken at magnifications of ×100 (**a**,**d**,**g**), ×400 (**b**,**e**,**h**), and ×1000 (**c**,**f**,**i**). (**B**) XRD patterns of PCL porous scaffold before and after treatments of irradiation and hydrolysis. The marks ◆, ▼, and ● indicate the representative patterns of PCL, α-TCP, and hydroxyapatite, respectively. (**C**) XPS chart of PCL porous scaffold after hydrolysis treatment. (**D**) ATR-FTIR spectrum of PCL porous scaffold after hydrolysis treatment.

Hydroxyapatite coating onto the PCL porous scaffold by the Er: YAG PLD method includes two treatments. One is the irradiation of the Er: YAG pulsed laser with a target of α-TCP to deposit the α-TCP microparticle onto the PCL fiber, and the other is hydrolysis, which induces the structural conversion from α-TCP to hydroxyapatite to finally obtain the hydroxyapatite-coated PCL. To confirm successful coating of hydroxyapatite onto the PCL porous scaffold, the surface state of fiber in the scaffold before and after each treatment was evaluated by several methods (Figure 1). It was observed by scanning electron microscope (SEM) that the PCL fiber before treatment has a smooth surface (Figure 1A(a–c)), while microparticles were homogenously deposited onto the surface of the fiber after the irradiation treatment (Figure 1A(d–f)). After the hydrolysis treatment, the appearance of microparticles was changed from a blocky structure to a needle-shaped one (Figure 1A(g–i)). On the other hand, the porous structure was maintained even after treatments of irradiation and hydrolysis. It was revealed by x-ray diffraction (XRD) that the representative patterns of α-TCP (JCPDS NO.09-0348) and hydroxyapatite (JCPDS NO.72-1243) emerged after treatments of irradiation and hydrolysis, respectively (Figure 1B). X-ray photoelectron spectroscopy (XPS) after the hydrolysis treatment detected calcium phosphate-derived peaks (O1s, Ca2p and P2p), while the Ca/P ratio was calculated to be 1.44 ± 0.02, which is comparable with the Ca/P ratio of calcium-deficient hydroxyapatite, one of the hydroxyapatite types (Figure 1C). Attenuated total reflection-Fourier transform infrared spectroscopy (ATR-FTIR) demonstrated that the peaks derived from PCL (1732 cm^−1^ (C = O stretching vibration), 2960 and 2869 cm^−1^ (C-H stretching vibrations)), -PO_4_^3−^ (1083, 1030, 962, 603, and 559 cm^−1^), and -OH^−^ groups (3574 and 630 cm^−1^) were detected after the hydrolysis treatment (Figure 1D). These results indicate that the hydroxyapatite coating onto the PCL porous scaffold was successfully achieved by the Er: YAG PLD method. The PCL porous scaffold coated with hydroxyapatite by the Er: YAG PLD method is expressed as “HAp-PCL” in the following experiments.

### 2.2. Mechanical and Surface Properties of PCL-Based Discs/Scaffolds

**Figure 2:**
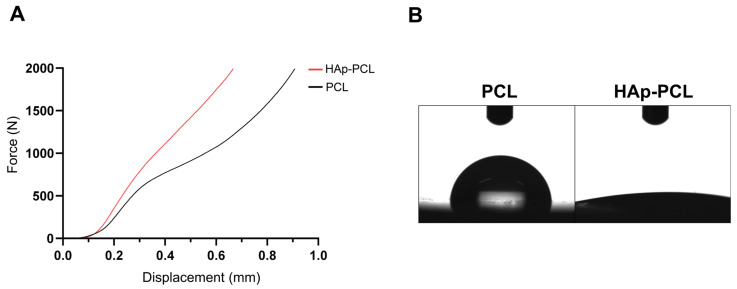
Mechanical and physicochemical surface properties of PCL-based discs/scaffolds. (**A**) Compressive strain curves of PCL and HAp-PCL scaffolds. (**B**) Images of water droplet on the surface of PCL and HAp-PCL discs.

Figure 2A shows the compressive strain curves of PCL and HAp-PCL scaffolds. The mechanical property of the PCL porous scaffold was reinforced by the hydroxyapatite coating. Figure 2B shows the images of a water droplet on the surface of PCL or HAp-PCL discs. The water wettability was drastically changed by the hydroxyapatite coating. The contact angles of PCL and HAp-PCL discs were 83.25 ± 0.86° and 6.86 ± 0.18°, respectively.

### 2.3. Behavior of Stem Cells after Seeding on PCL-Based Discs/Scaffolds

**Figure 3:**
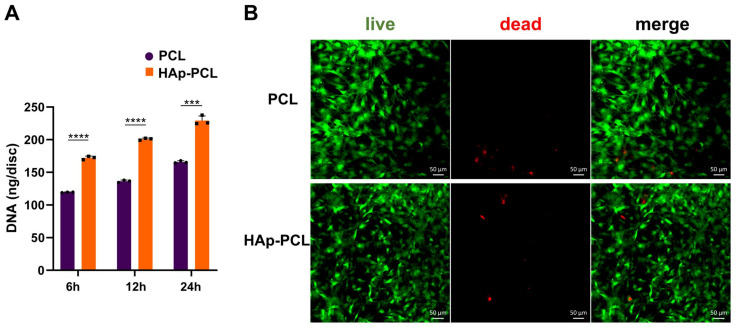
Assessment of initial cell attachment and cell viability after seeding on PCL-based discs. (**A**) Initial attachment of rBMSCs after seeding on discs of PCL or HAp-PCL. *** *p* < 0.001, **** *p* < 0.0001: significant difference between two groups at the corresponding time. (**B**) Viability of rBMSCs cultured on discs of PCL or HAp-PCL for 24 h, evaluated by the live/dead assay. Scale bar is 50 μm.

Rat bone marrow-derived mesenchymal stem cells (rBMSCs) were used to explore the behavior of cells for the PCL-based discs/scaffolds. Figure 3A shows the initial attachment of rBMSCs on disc of PCL with or without hydroxyapatite coating. The number of cells attached onto the HAp-PCL disc was significantly higher than that on the PCL disc. In addition, the cell viability was also evaluated by the live/dead assay (Figure 3B). Most of the rBMSCs were viable on PCL discs, irrespective of hydroxyapatite coating.

**Figure 4:**
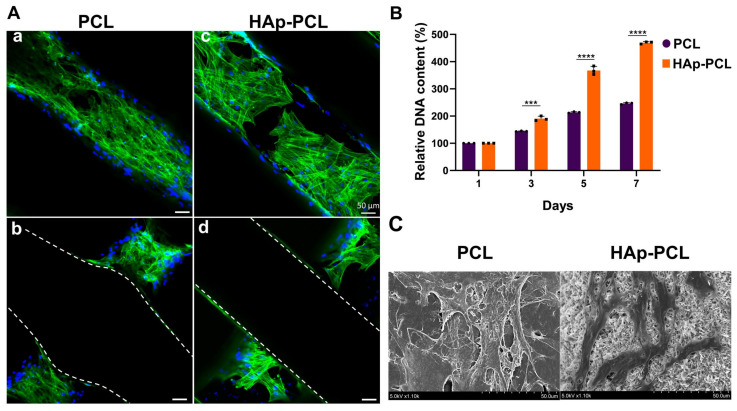
The behavior of rBMSCs on PCL-based scaffolds. (**A**) Fluorescent microscopic images of rBMSCs 24 h after culturing on PCL (**a**,**b**) or HAp-PCL (**c**,**d**). Images were acquired in the outermost (**a**,**c**) and interior (**b**,**d**) planes. The region between the dashed lines indicates a fiber of the scaffold. Actin filaments and nucleus were stained and are indicated in green and blue, respectively. Scale bar is 50 μm. (**B**) Time profiles of relative DNA content of BMSCs cultured on PCL or HAp-PCL. *** *p* < 0.001, **** *p* < 0.0001: significant difference between two groups at the corresponding time. (**C**) SEM images of rBMSCs cultured on PCL or HAp-PCL for 3 days.

The attachment behavior of rBMSCs on PCL porous scaffold with or without hydroxyapatite coating was evaluated by the visualization of actin filaments (Figure 4A). The actin filaments were clearly observed for rBMSCs attached on both scaffolds, while cells were attached on the fiber of the scaffold, which is located not only on the surface (Figure 4A(a,c)) but also on the interior of the scaffold (Figure 4A(b,d)). The DNA content was chronologically measured to evaluate the proliferative activity of rBMSCs in each scaffold after seeding (Figure 4B). The DNA content of both scaffolds gradually increased with time, while the proliferation rate of rBMSCs on HAp-PCL was higher compared with that on PCL. Figure 4C shows the SEM images of the cells cultured on each scaffold. The rBMSCs were attached and spread well on both scaffolds. In the HAp-PCL group, the pseudopods of cells entering into the hydroxyapatite microstructure were observed.

### 2.4. Bone Formation by the Implantation of PCL-Based Scaffolds

**Figure 5:**
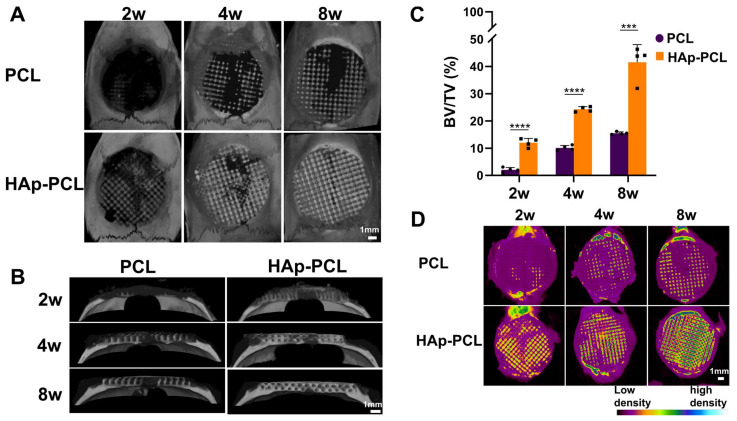
μ-CT analysis of bone formation at 2, 4, and 8 weeks after implantation of PCL or HAp-PCL into critical-sized calvaria bone defects in rats. The μ-CT images were acquired from the axial (**A**) and coronal (**B**) planes of the defective site. (**C**) Time profiles of bone volume/total volume (BV/TV). The bone volume for the HAp-PCL group were calculated by subtracting the value of scaffold-derived hydroxyapatite from that of the original signal. (**D**) Bone mineral density (BMD) images obtained from the axial plane of μ-CT. *** *p* < 0.001, **** *p* < 0.0001: significant difference between two groups at the corresponding time.

To evaluate the potential of bone regeneration, two types of PCL-based scaffolds (PCL and HAp-PCL) were implanted into the critical-sized calvaria bone defect in F344 rats, as previously used [34,35]. In this study, a negative control group (no implantation) was omitted because little bone formation without any implantation was previously confirmed, and the effect of hydroxyapatite coating on the bone formation efficacy by PCL porous scaffold was focused. The bone formation was evaluated by micro-computed tomography (μ-CT) analysis (Figure 5). The increment of the signal based on bone formation was observed in the pores of both scaffolds, while the signal intensity gradually increased with time (Figure 5A). In the HAp-PCL group, signal increment occurred, even in the center of the scaffold until 8 weeks after implantation (Figure 5B). The percentage of bone volume to tissue volume for the HAp-PCL group was significantly higher than that for the PCL group (Figure 5C). In addition, bone with high mineral density was observed in the HAp-PCL group, while the value increased with time (Figure 5D).

### 2.5. Histological Evaluation of Bone Formation by the Implantation of PCL-Based Scaffolds

**Figure 6:**
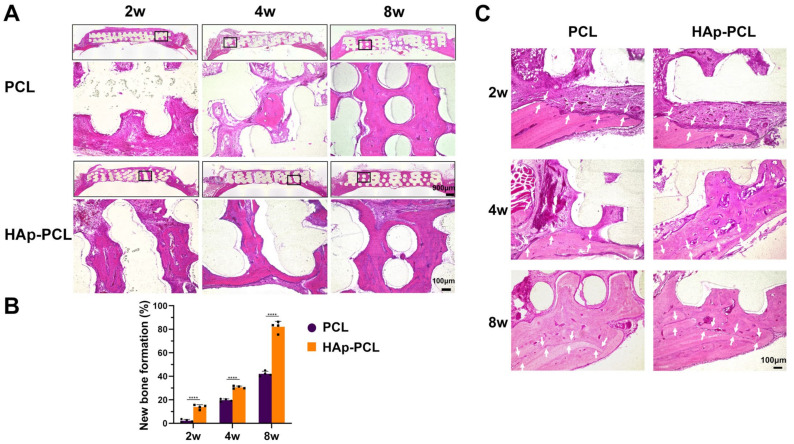
H&E staining for the sectioned tissue inside the scaffold at 2, 4, and 8 weeks after implantation of PCL or HAp-PCL into the critical-sized calvaria bone defect in rats. (**A**) Representative staining images. The frames in the upper image indicate the region of lower image with high maginification. Scale bars are 100 (lower images) or 900 μm (upper images). (**B**) Percentage of new bone area formed analyzed by the staining results. **** *p* < 0.0001: significant difference between two groups at the corresponding time. (**C**) Staining images of tissue surrounding the implant-host bone interface. The implant-host bone interface is present between two arrows. Scale bar is 100 μm.

Several histological evaluations were performed in the bone formation induced by the implantation of PCL-based scaffolds (PCL and HAp-PCL). Hematoxylin and eosin (H&E) staining was performed to explore the condition of tissue surrounding the bone defect (Figure 6A). No inflammatory reaction caused by the implantation of both scaffolds was observed. In the case of the PCL group, most of the pores in the PCL were filled with connective tissue 2 weeks after implantation, then partial bone formation was observed 8 weeks after implantation. On the other hand, in the HAp-PCL group, bone tissue was observed in the defective side only 2 weeks after implantation. The pores in the center region of the scaffold were almost filled with bone tissue until 8 weeks after implantation. The extent of bone tissue regenerated by the implantation of HAp-PCL was significantly higher than that by PCL at corresponding time periods (Figure 6B). Figure 6C shows the histological images of tissue surrounding the implant-host bone interface. The interface between the implant and the host bone seemed to disappear with time for both PCL and HAp-PCL groups, while the disappearance rate for the HAp-PCL group was faster than that for the PCL group. This result indicates that the HAp-PCL implant well integrated with the surrounding host bone tissue with time.

**Figure 7:**
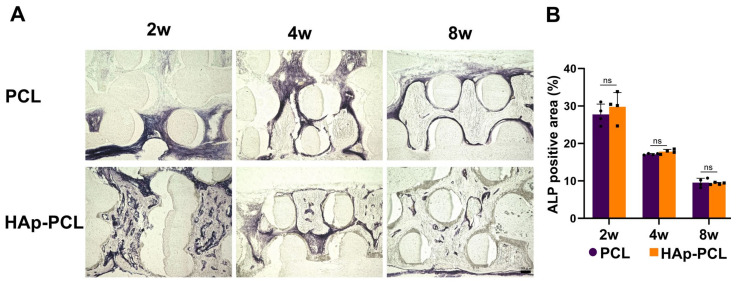
Alkaline phosphatase (ALP) staining for the sectioned tissue inside the scaffold at 2, 4, and 8 weeks after implantation of PCL or HAp-PCL into the critical-sized calvaria bone defect in rats. (**A**) Representative staining images. Scale bar is 100 μm. (**B**) Time profiles in percentages of ALP-positive area. n.s.; no significance.

Alkaline phosphatase (ALP) staining was performed to visualize the presence of osteoblasts surrounding the bone defect after the implantation of each scaffold (Figure 7A). The distribution of ALP-positive cells in the tissue was clearly different between the two groups 2 weeks after implantation. In the PCL group, the ALP-positive cells were detected near the defective side of the scaffold. On the other hand, in the HAp-PCL group, ALP-positive cells were present throughout the scaffold and formed an aggregated structure. There was no difference in the percentage of ALP-positive area between the two groups at the corresponding time, while the ALP-positive area decreased with time in both groups. (Figure 7B).

**Figure 8:**
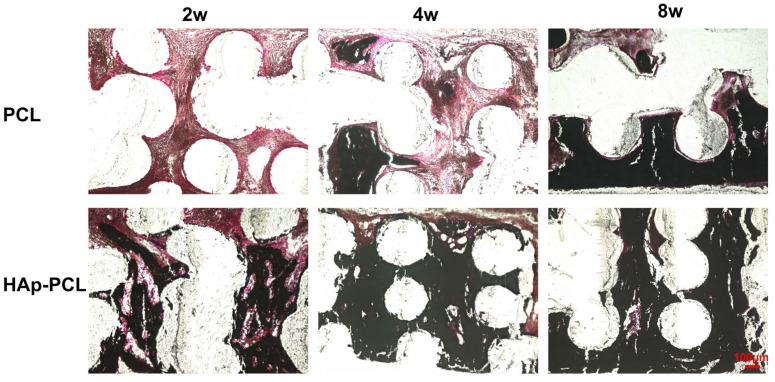
Von Kossa staining for the sectioned tissue inside the scaffold at 2, 4, and 8 weeks after implantation of PCL or HAp-PCL into the critical-sized bone defect in rats. The tissues were counterstained with Nuclear Fast Red solution. Scale bar was 100 μm.

Von Kossa staining was performed to visualize the calcium deposition in the tissue surrounding the bone defect after the implantation of each scaffold (Figure 8). In the PCL group, the calcium was gradually deposited from the defective side of the scaffold with time. On the other hand, in the HAp-PCL group, much more calcium deposition in the entire scaffold was observed only 2 weeks after implantation.

**Figure 9:**
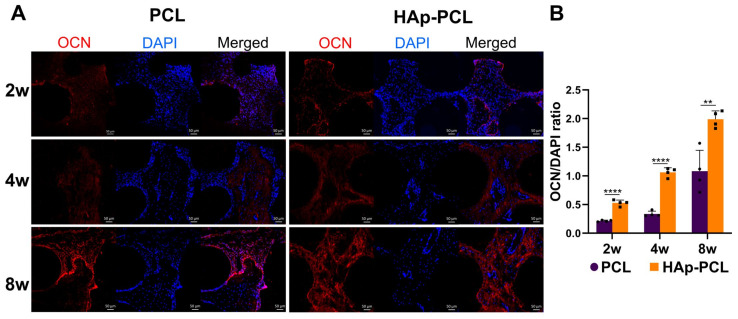
Immunohistochemical staining of cytosolic osteocalcin (OCN, red) for the sectioned tissue inside the scaffold at 2, 4, and 8 weeks after implantation of PCL or HAp-PCL into the critical-sized bone defect in rats. (**A**) Representative staining images. The cell nucleus (blue) was stained with 4’,6-diamidino-2-phenylindole (DAPI). Scale bar is 50 μm. (**B**) Time profiles in the ratio of osteocalcin-positive area. ** *p* < 0.01, **** *p* < 0.0001: significant difference between two groups at the corresponding time.

Immunohistochemical staining for osteocalcin (OCN) was performed to evaluate the maturation of osteoblast surrounding the bone defect after the implantation of each scaffold. A decreased number of cells and an increased OCN-positive area were observed as the bone formation proceeded, especially in the HAp-PCL group (Figure 9A). The ratio of OCN-positive area to nucleus number for the HAp-PCL group was significantly higher than that for the PCL group (Figure 9B).

**Figure 10:**
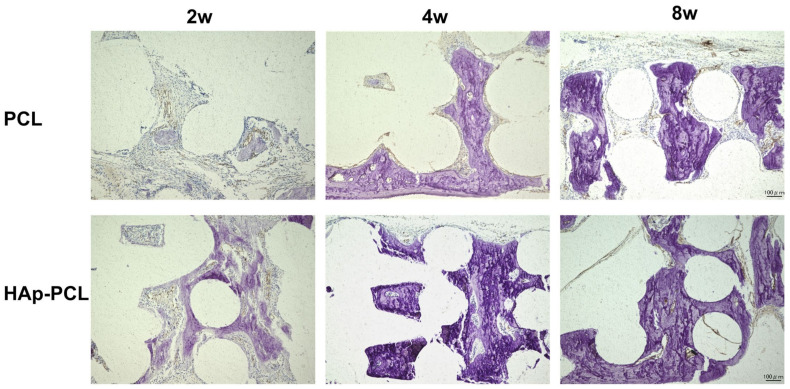
Immunohistochemical staining of von Willebrand factor (brown) for the sectioned tissue inside the scaffold at 2, 4, and 8 weeks after implantation of PCL or HAp-PCL into the critical-sized bone defect in rats. The tissues were counterstained with hematoxylin (purple). Scale bar was 100 μm.

Vascularization of the scaffold after implantation was also evaluated (Figure 10). In both scaffolds, the neovascularization occurred in the pore of the scaffold. There is no difference in the degree of vascularization between the PCL and HAP-PCL groups. This result indicates that the hydroxyapatite coating did not influence vascularization in the implantation site.

## 3. Discussion

The present study demonstrates that early bone formation for a large defect was achieved by the hydroxyapatite coating of PCL porous scaffold through the Er: YAG PLD method. When implanted into the critical-sized calvaria bone defect in rats, the PCL porous scaffold coated with hydroxyapatite induced not only the rapid infiltration of endogenous cells into the scaffold based on the geometry and surface chemistry, but also the continuous interaction of hydroxyapatite with cells, resulting in early bone formation.

In this study, the Er: YAG PLD method was used for the hydroxyapatite coating of PCL porous scaffold. The Er: YAG PLD is composed of an Er: YAG pulsed laser and a target to be deposited. Er: YAG PLD is achieved by the transpiration of a hydration shell around the target through the instantaneous absorption of the laser energy, the ablation of micro crystalline particles from the target, and deposition onto the substrate. An advantage of the Er: YAG PLD method over deposition methods using other laser sources is that the deposition can be achieved under atmospheric and ambient conditions. In previous studies, α-TCP deposition has been successfully achieved directly on the surface of titanium plate and demineralized bovine enamel using Er: YAG-PLD. In addition, the coating of hydroxyapatite thin film has been obtained by the combination with hydrolysis-based structural conversion from α-TCP to hydroxyapatite [29,30]. There is no report on the application of the Er: YAG-PLD method for the preparation of polymer-based composite.

The SEM image demonstrated that hydroxyapatite coating was achieved by the Er: YAG-PLD method, maintaining the porous structure (Figure 1A). From the analyses of XPS and ATR-FTIR (Figure 1C,D), it is possible that the component obtained by the Er: YAG-PLD method and the subsequent hydrolysis is calcium-deficient hydroxyapatite, one of the hydroxyapatite types. It is known that the composition of hydroxyapatite in the living system (bone mineral, dentin, or enamel) is not identical in the aspect of stoichiometry to that obtained by chemical synthesis (Ca/P ratio = 1.67) [36,37]. Although the calcium-deficient hydroxyapatite is not as stable as general hydroxyapatite in the physiological condition, its biological properties are similar to the bone component, which plays a very important role in bone formation [38,39].

For efficient bone formation in the defective tissue, it is necessary to develop a scaffold that possesses the moderate mechanical strength that allows endogenous cells to infiltrate, attach, proliferate, and differentiate into osteocytes. It is revealed that the hydroxyapatite coating improved the mechanical strength against compression (Figure 2A). The improved mechanical strength of the PCL scaffold is due to the dense presence of hydroxyapatite with higher stiffness compared with PCL of polymer. The improved mechanical property implies that the scaffold can provide mechanical support for tissue reconstruction during the process of the degradation of PCL during bone formation and its eventual replacement by fully functional tissue. In addition, it is also shown that the hydroxyapatite coating improved water wettability (Figure 2B). The enhanced water wettability of the PCL scaffold after coating is also owing to the presence of hydroxyapatite with a hydrophilic nature. Considering that the porous structure was maintained after the hydroxyapatite coating (Figure 1A), the improvement contributes to good infiltration of endogenous cells into the interior of porous scaffolds, maintaining the transport properties of nutrient input and waste removal, which supports continuous tissue growth. It was clearly demonstrated that rBMSCs could adhere and show a viability on PCL or HAp-PCL discs (Figure 3). One of the reasons why the number of rBMSCs attached onto the HAp-PCL was significantly higher than that on PCL was considered to be suitable water wettability for cell attachment. The rBMSCs were also homogenously adhered and proliferated on the porous scaffold of PCL or HAp-PCL (Figure 4).

Surprisingly, partial bone formation was observed for the implantation of PCL without a hydroxyapatite coating into the critical-sized calvarial bone defect in rats (Figure 5 and Figure 6). This may be due to the porous structure being geometrically suitable for the infiltration of endogenous cells. The bone formation was accelerated by the hydroxyapatite coating. There are two reasons for the accelerated bone formation. One is the improvement of water wettability. The combination of suitable porosity and water wettability enables endogenous cells related to the bone regeneration to rapidly infiltrate into the interior of the porous scaffold. The other is the biological functions of hydroxyapatite coated on PCL. A large number of studies have now demonstrated the intrinsic ability of hydroxyapatite to promote osteogenic differentiation of stem cells in vitro [21,40,41,42,43]. It has been suggested that the nanostructured hydroxyapatite can induce the osteogenic differentiation of stem cells and form the new bone through in vivo bio-mineralization by the release of ions (Ca^2+^ and PO_4_^3^^−^) by degradation [44]. The behavior of hydroxyapatite in degradation–diffusion–reconstruction during bone repair has also been visualized [20]. The degradation–diffusion–reconstruction behavior of hydroxyapatite shows the dynamics of hydroxyapatite in the process of bone repair. When implanted, the hydroxyapatite degraded into ions of Ca^2+^ and PO_4_^3−^. Then, the Ca^2+^ and PO_4_^3−^ released from hydroxyapatite are diffused to induce the migration and proliferation of osteoblasts. The Ca^2+^ and PO_4_^3−^ also induce the osteogenic differentiation of osteoblasts or stem cells to reconstruct the bone tissue. Considering these events and the staining result (Figure 7, Figure 8 and Figure 9), it seems that the degradation–diffusion–reconstruction behavior occurred within 2 weeks after implantation. It is recognized that the chemical properties and physical characteristics of calcium phosphate, such as solubility, microporosity, and roughness, can lead to different rates of bone regeneration [45,46]. As described above, one type of hydroxyapatite to be coated could be calcium-deficient hydroxyapatite. Because the bone regeneration rate of calcium-deficient hydroxyapatite is high [38,39,47], the data for accelerated bone formation obtained in this study agreed with previous report.

It is well known that osteoblasts are involved during bone formation [48]. Initially, osteoblasts with the marker of alkaline phosphatase emerged in the defective site. The osteoblasts produce the osteoid matrix to induce mineralization and are matured to express osteocalcin. The results of histological staining (Figure 7, Figure 8 and Figure 9) were consistent with these events. In the PCL group, these cellular events were limited to occurring only near the defective side of the scaffold at the early phase (2 weeks after implantation). In the HAp-PCL group, the events had already occurred throughout the scaffold at the early phase. Taken together, the difference in the dynamics of osteoblasts affects the subsequent bone formation.

There are several limitations in this study. The complete degradation of PCL was not observed in this study. The retarded degradation physically interferes with the ideal tissue regeneration. It is necessary to evaluate and optimize the degradation behavior of the materials used for scaffold preparation in the future. In addition, the detailed mechanism of bone formation in terms of cellular dynamics remains unclear at present. How the osteoblast or vascular endothelial cells emerge in the scaffold still needs to be clarified in future investigations.

## 4. Materials and Methods

### 4.1. Hydroxyapatite Coating onto the PCL Porous Scaffold by the Er: YAG-PLD Method

A α-TCP bulk (cylinder with a diameter and height of 5 and 7 mm, respectively) prepared by a hydraulic press (0.3 MPa) of α-TCP powder (Taihei Chemical Industrial Co., Ltd., Osaka, Japan) was used as a target for the irradiation of the Er: YAG laser. An Er: YAG laser unit, equipped with a straight laser tip, C400F (Erwin AdvErl Unit; Morita Manufacturing Corp., Kyoto, Japan), was used for the PLD. The target was moistened with pure water, and the laser tip was positioned as close to the target as possible. Pulsed laser deposition was performed for the PCL porous scaffold (3D Insert-PCL, disc shape (10 mm diameter, 1.5 mm height), fiber diameter: 300 μm, pore: 300 μm, 3D Biotek, Bridgewater, NJ, USA) at a laser output of 300 mJ at 3 pps, and the handpiece was moved manually to obtain a uniform α-TCP deposition. Irradiation was made for both sides of the PCL, and the irradiated scaffolds were immediately immersed in deionized water at 45 °C for 3 days for the hydrolysis.

### 4.2. Characterization of PCL Porous Scaffolds Treated by the Er: YAG-PLD Method

After sufficient drying by air, the PCL porous scaffolds before and after treatments of irradiation and hydrolysis were sputter-coated with osmium and observed by scanning electron microscope (SEM) (S-4800; Hitachi High-Tech Corp., Tokyo, Japan). The crystal structures of the scaffolds before and after treatments of irradiation and hydrolysis were investigated using the X-ray diffraction system (XRD-6000, Shimadzu Co., Tokyo, Japan) with Cu Kα radiation at 40 kV and 30 mA. The scanning speed was 2°/min and the 2θ range was 10–70°. The composition of element and chemical states on the surface of a PCL porous scaffold after treatment of hydrolysis was measured by X-ray photoelectron spectrometry (ESCA-5600; ULVAC-PHI, Kanagawa, Japan). ATR-FTIR (IRAffinity-1S, Shimadzu Co., Kyoto, Japan) was used to analyze chemical bonding in the PCL porous scaffold after hydrolysis treatment. The measurement was carried out at wavenumbers ranging from 400 to 4000 cm^−1^, while the number of scans was 16.

### 4.3. Evaluation of Mechanical and Physicochemical Properties of PCL-Based Discs/Scaffolds

Each scaffold (PCL or HAp-PCL) was pressed with the precision universal test machine (AGS-X, Shimadzu Co., Kyoto, Japan) at a crosshead speed of 0.5 mm/min, and the forces at the designated strains were recorded. Three scaffolds for each group were used for the force-strain measurement. A PCL porous scaffold was placed on the slide glass and warmed at 60 °C with the hot plate and pressed using the cover glass to obtain a PCL disc without any pores on the surface. The hydroxyapatite coating of PCL discs was performed by the same procedure as that of the PCL porous scaffold by the Er: YAG PLD method described above. One drop of distilled water (7 μL) was added on each disc (PCL or HAp-PCL) at room temperature, and the contact angle was measured by the wettability evaluation device (LSE-ME2; Nick Corporation, Saitama, Japan). Three discs for each group were used for the measurement of contact angle.

### 4.4. In Vitro Cell-Based Studies

#### 4.4.1. Initial Attachment and Viability of Cells on PCL-Based Discs

Each disc of PCL or HAp-PCL was sterilized by a fully automated ethylene oxide gas sterilizer (SA-N160, Iki Co., Otsu, Japan). The rBMSCs were collected and cultured from the tibia and femur of 6-week-old male F344 rats according to the protocol in the previous article [49], and cells with the passage numbers of 3 and 4 were used for in vitro cell-based studies. The rBMSCs were cultured with α-MEM (FUJIFILM Wako Co., Osaka, Japan) containing 20% fetal bovine serum (FBS, Cytiva, Tokyo, Japan) and 1% antibiotics and antimycotics (Gibco Invitrogen, Carlsbad, CA, USA). The cells (3 × 10^4^ cells) were seeded onto each disc (PCL or HAp-PCL), and the discs were placed in a 24-well plate. The DNA concentration of cells 6, 12, and 24 h after seeding were measured using the Quant-iT™ PicoGreen™ dsDNA Assay Kit (Thermo Fisher Scientific Inc., Waltham, MA, USA) according to the manufacturer’s instruction. After seeding rBMSCs on each disc (PCL or HAp-PCL) for 24 h, viability was assessed using the LIVE/DEAD Viability/Cytotoxicity Kit (Thermo Fisher scientific Inc., Waltham, MA, USA) according to the manufacturer’s instruction. The stained cells were observed by confocal laser fluorescence microscope (LSM-700, Zeiss Microscopy, Jena, Germany). Each experiment was performed in triplicate.

#### 4.4.2. Adhesion and Proliferation of Cells on PCL-Based Scaffolds

The suspension of rBMSCs (50 μL, 2 × 10^5^ cells) was seeded onto each scaffold (PCL or HAp-PCL) and the scaffolds were placed in a 24-well plate and incubated for 3 h. Then, the medium was slowly added from the side wall of the 24-well plates. The plate was returned to the incubator after ensuring that the scaffolds were completely soaked and settled to the bottom of the wells. After 24 h incubation, the scaffolds with cells were washed with phosphate-buffered saline (PBS) and fixed with 4% paraformaldehyde (PFA) for 30 min at room temperature. The scaffolds were washed four times with PBS, and cells were permeabilized with 0.2% Triton-X-100 (Sigma-Aldrich, Burlington, MA, USA) solution for 30 min. The Phalloidin-iFluor™ 488 Conjugate (Cayman Chemical Co., Ann Arbor, MI, USA) and DAPI solution (AAT Bioquest Inc., Sunnyvale, CA, USA) was added and incubated for 60 min to allow cells to specifically bind to the actin filaments in the cells. The cells were then washed 5 times with PBS and the cell adhesion in each scaffold was observed by confocal laser scanning microscope. The DNA contents of rBMSCs at 1, 3, 5, and 7 days after seeding were assessed with the Quant-iT™ PicoGreen™ dsDNA Assay Kit. The medium was replaced three times per week. The cell morphology in each scaffold was observed by scanning electron microscopy (SEM). The scaffolds with cells were fixed in 4% PFA for 1 h at room temperature, washed with PBS, dehydrated with ethanol of sequentially increasing concentrations (20%, 40%, 60%, and 80% immersed for 3 min each, followed by 100% immersed for 2 min), and sputter-coated with osmium for SEM analysis.

### 4.5. Bone Formation by the Implantation of PCL-Based Scaffolds

#### 4.5.1. Animal Experiments

F344 rats (male, 8-week-old) were purchased from Shimizu Laboratory Supplies Co., Kyoto, Japan. Preoperative anesthesia was performed by the intraperitoneal injection of a mixture of three anesthetic agents (butorphanol tartrate: 2.5 mg/kg, midazolam: 2 mg/kg, and medetomidine hydrochloride: 0.15 mg/kg). A critical-sized bone defect was created in the calvaria using a 9 mm diameter trephine bar (Micro Tech Co, Tokyo, Japan) and a handpiece (FX23; Nakanishi Inc., Tochigi, Japan) while cooling with sterile saline solution. Each scaffold (PCL or HAp-PCL) was implanted into the bone defect. Four rats were used for each group. After that, the periosteum and skin were covered and tightly sutured to prevent them from movement. At 2, 4, and 8 weeks after implantation, the rats were euthanized and the tissues surrounding the calvaria bone defect were collected. The tissues collected were fixed with 4% paraformaldehyde phosphate buffer solution (FUJIFILM Wako Pure Chemical Co., Osaka, Japan) and preserved at 4 °C.

#### 4.5.2. Microcomputed Tomography (μCT)

Fixed samples were scanned with the μCT (SKYSCAN 1275, Bruker Co., Billerica, MA, USA) at 65 kV and 85 μA, and bone formation was quantitatively evaluated with the analysis software (CTAn, Bruker Co., Billerica, MA, USA) to obtain the bone volume fraction (Bone volume/Tissue volume (BV/TV)) and the axial plane bone mineral density (BMD) images. The bone volumes for the HAp-PCL group were calculated by subtracting the value of the scaffold-derived HAp from that of the original signal.

#### 4.5.3. Histological Analysis

The fixed samples were divided into two parts; one part was decalcified for 5 days with Decalcifying Solution B (FUJIFILM Wako Pure Chemical Co., Osaka, Japan) and the other part was used without any decalcification. By the Kawamoto method [50], frozen sections with a thickness of 7 μm were prepared for all samples. The sections from decalcified samples were stained with hematoxylin and eosin. The sections from non-decalcified specimens were subjected to Von Kossa staining, ALP staining, and immunohistochemical staining for vWF and OCN.

For the ALP staining, an ALP Stain Kit (FUJIFILM Wako Pure Chemical Co., Osaka, Japan) was used. The sections were washed with PBS, followed by adding 0.3 mL of ALP substrate solution for 30 min in a moist chamber at room temperature. The sections were then washed three times with distilled water, and a nuclear staining solution (0.5 mL) was added to the sections. After 4–5 s, the sections were washed with distilled water. The sections were dehydrated, soaked in xylene, and finally mounted with Glycerol (FUJIFILM Wako Pure Chemical Co., Osaka, Japan).

For Von Kossa staining, a Calcium Stain Kit (ScyTek Laboratories Inc., West Logan, UT, USA) was used. The sections were washed with PBS and incubated with Sliver Nitrate Solution for 30 min while being exposed to ultraviolet light. Then, the sections were washed with distilled water and incubated in Sodium Thiosulfatem Solution for 2 min. The sections were washed and stained with Nuclear Fast Red Solution for 5 min. The sections were washed, dehydrated in absolute alcohol, and mounted in DPX new (Sigma-Aldrich, Burlington, MA, USA). The image of the stained sections was acquired with a digital microscope (BZ-9000; Keyence Corp., Osaka, Japan).

For vWF immunostaining, the antigen molecules of sections were activated with Dako Proteinase K Ready-to-use (S3020, Agilent Technologies, Inc., Santa Clara, CA, USA) and blocked with a blocking solution (Dako Protein Block Serum-Free Ready-to-use; Agilent Technologies, Inc., Santa Clara, CA, USA). The anti-Von Willebrand Factor antibody (ab6994, Abcam, Cambridge, UK) was used at a dilution of 1:1000 and Dako EnVision+ System- HRP Labelled Polymer Anti-Rabbit (K4003, Agilent Technologies, Inc., Santa Clara, CA, USA) was used to achieve qualitative identification of antigens by light microscopy.

The metrological analysis from acquired image was performed using ImageJ version 1.53 (National Institutes of Health, Bethesda, MD, USA). The percentages of new bone formation in the scaffold and ALP-positive area were calculated by the following equations:

New bone formation (%) = New bone area/Space area available in the scaffold × 100

ALP-positive area (%) = ALP-positive area/Space area available in the scaffold × 100

For immunofluorescence staining, antigens in the sections were first activated with an antigen activation solution (NACALAI TESQUE, Inc., Kyoto, Japan). After washing, the sections were blocked and permeabilized with 5% goat serum and 0.1% Triton X-100 in PBS, respectively. Then, the sections were stained with Rabbit Anti-Rat Osteocalcin Polyclonal Antibody conjugated with ALEXA FLUOR^®^ 647 (bs-4917R-A647, Bioss Antibodies Inc., Woburn, MA, USA) at a dilution of 1:100 and incubated overnight at 4 °C. Finally, the sections were washed with Tris-buffered saline containing 0.1% Tween 80 and mounted on DAPI-Fluoromount-G^®^ (Southern Biotechnology Associates Inc., Birmingham, AL, USA). Images were taken using a confocal fluorescence microscope (LSM-700), and the ratio of OCN-positive area (OCN-positive area/DAPI area × 100) at the corresponding time were evaluated using ImageJ ver. 1.53.

### 4.6. Statistical Analysis

All data were expressed as mean ± standard deviation. All data were analyzed statistically using GraphPad Prism 8 (GraphPad Software Inc., San Diego, CA, USA). The Student’s *t*-test was carried out to determine the statistical differences between the two groups at each time point. 

## 5. Conclusions

The hydroxyapatite coating of PCL porous scaffolds was achieved by the Er: YAG pulsed laser deposition method. By the hydroxyapatite coating, the PCL porous scaffold gained a suitable mechanical property and water wettability. In vitro experiments demonstrated that the initial attachment, viability, and proliferation of cells in the scaffold was improved by the hydroxyapatite coating. When implanted into the critical-sized calvaria bone defect in rats, the PCL porous scaffold coated with hydroxyapatite demonstrates accelerated and widespread bone formation. Taken together, the PCL porous scaffold coated with hydroxyapatite obtained by the Er: YAG pulsed laser deposition method is a promising material in bone tissue engineering.

## Data Availability

Not applicable.
